# Anti-Inflammatory Effect of Supercritical-Carbon Dioxide Fluid Extract from Flowers and Buds of *Chrysanthemum indicum* Linnén

**DOI:** 10.1155/2013/413237

**Published:** 2013-10-08

**Authors:** Xiao-Li Wu, Chu-Wen Li, Hai-Ming Chen, Zu-Qing Su, Xiao-Ning Zhao, Jian-Nan Chen, Xiao-Ping Lai, Xiao-Jun Zhang, Zi-Ren Su

**Affiliations:** ^1^School of Chinese Materia Medica, Guangzhou University of Chinese Medicine, Waihuandong Road No. 232, Guangzhou Higher Education Mega Center, Guangzhou 510006, China; ^2^Dongguan Mathematical Engineering Academy of Chinese Medicine, Guangzhou University of Chinese Medicine, Songshan Lake High-Tech Industrial Development Zone, Dongguan, Guangdong 523808, China

## Abstract

The aim of this study was to analyze the chemical composition and investigate the anti-inflammatory property of the supercritical-carbon dioxide extract from flowers and buds of *C. indicum* (CI_SCFE_). The anti-inflammatory effect was evaluated in four animal models including xylene-induced mouse ear edema, acetic acid-induced mouse vascular permeability, carrageenan-induced mouse hind paw edema, and cotton pellet-induced rat granuloma formation. The results indicated that CI_SCFE_ significantly attenuated xylene-induced ear edema, decreased acetic acid-induced capillary permeability, reduced carrageenan-induced paw, and inhibited the cotton pellet-induced granuloma formation in a dose-dependent manner. Histopathologically, CI_SCFE_ abated inflammatory response of the edema paw. Preliminary mechanistic studies demonstrated that CI_SCFE_ decreased the MDA level via increasing the activities of anti-oxidant enzymes (SOD, GPx, and GRd), attenuated the productions of NF-**κ**B, TNF-**α**, IL-1**β**, IL-6, PGE_2_ and NO, and suppressed the activities of iNOS and COX-2. In phytochemical study, 35 compounds were identified by GC-MS, and 5 compounds (chlorogenic acid, luteolin-7-glucoside, linarin, luteolin and acacetin) were reconfirmed and quantitatively determined by HPLC-PAD. This paper firstly analyzed the chemical composition by combining GC-MS with HPLC-PAD and explored possible mechanisms for the anti-inflammatory effect of CI_SCFE_.

## 1. Introduction

Inflammatory reaction, typically characterized by redness, swelling, heat, and pain, is a common physiologic response to defend the host from injurious stimuli such as pathogens, toxins and local injuries [1]. Thus, inflammation has been regarded as a protective attempt to eliminate the harmful stimuli and to activate the healing process by the organism. However, persistent or overinflammation leads to tissue damage and possibly failure of organs. Nowadays, nonsteroidal anti-inflammatory drugs (NSAIDs) are the most commonly prescribed therapeutics for inflammatory diseases [2]. But, owing to the adverse side effects, such as gastrointestinal ulcers, bleedings, and renal damages induced by long-term administration, the use of NSAIDs is becoming highly controversial [3]. Therefore, increasing attention has been focused on ethnological medicinal plants as they are affordable and have less toxicities and adverse effects [4]. 

*Chrysanthemum indicum *Linnén (*C*. *indicum*) is a traditional Chinese medicine (TCM), which has been used to treat various inflammation-related diseases with high efficacy and low toxicity for several centuries [5, 6]. Modern pharmacological researches demonstrated that the ethanolic extract of *C*. *indicum* decreased inflammatory mediators in lipopolysaccharide- (LPS-) induced RAW 264.7 cells by suppressing nuclear factor kappa B- (NF-*κ*B-) dependent pathways [7] and attenuated mouse contact dermatitis through blocking synthesis of proinflammatory cytokines, such as nitrite oxide (NO) and prostaglandin E_2_ (PGE_2_) [8]. The phytochemical profiles of this plant showed the presence of flavonoids, terpenoids, phenolic compounds, and alkenes, some of which had been suggested to be responsible for its therapeutic properties against inflammation-related diseases via scavenging reactive oxygen species (ROS) and reducing proinflammatory cytokines [5, 6, 9–13].

Currently, the supercritical-carbon dioxide (CO_2_) fluid extraction (SCFE) technology has been successfully applied to the extraction process of *C*. *indicum* [14]. The SCFE of *C*. *indicum* (CI_SCFE_) has been widely used as a fine material in many TCM preparations (e.g., *C*. *indicum* granules and capsules), functional foods (e.g., tea bags and candies), cosmetics (e.g., facial masks and creams), and toiletries (e.g., toothpastes and mouth washes). Most importantly, the CI_SCFE_ is a main ingredient of a TCM recipe named Compound *C. indicum* Soft Capsule (also known as CPZ in previous studies), an anti-influenza product whose activity is closely related to its anti-inflammatory features [15, 16]. However, the chemical composition of CI_SCFE_ and whether it has anti-inflammatory activity are unknown so far. Thus, in this study, we analyzed the chemical ingredients of CI_SCFE_ by Gas Chromatography-Mass Spectrometry (GC-MS) analysis and High Performance Liquid Chromatography-Photodiode Array Detector (HPLC-PAD) analysis and examined the anti-inflammatory activity on various animal models.

It is well known that inflammation is a complex physiological response mediated by a variety of signaling molecules produced by leukocytes, macrophages, monocytes, mast cells, and activations of complement factors [17]. In general, when encountering harmful stimuli, acute inflammation is to be activated as the initial phase of inflammatory process, characterized by not only vascular changes including vasodilatation, permeability accentuation, and blood flow reduction but also edema formations resulting from extravasations of fluid and proteins at the inflammatory site [18]. These inflammatory processes are accompanied by neutrophil infiltrations, cytokine releases, and other pro-inflammatory mediator productions. In addition, activated neutrophils secrete multifarious proteases and generate ROS, both of which destroy invading particles and also damage cells and tissues of the host [19]. The acute inflammatory response will lead to chronic inflammation that is featured by tissue proliferation, granuloma, and repair. Nevertheless, the excessive inflammation can be harmful, even lethal, to the host [17, 20].

Xylene-induced mouse ear edema, acetic acid-induced mouse vascular permeability accentuation, carrageenan-induced mouse paw edema, and cotton pellet-induced rat granuloma are designed for examining the vasodilatation, vascular permeability, edema formation, and tissue hyperplasia, respectively [20]. In this paper, by using these models, we systematically investigated the anti-inflammatory property of CI_SCFE_ on acute inflammation and chronic inflammation. In particular, carrageenan-induced mouse paw edema model has been reported to be the most repeatable and reliable model to examing the interference of pharmaceutical agents on the proinflammatory mediators among various inflammation models [20, 21]. Therefore, we determined the levels of inflammatory factors in the edema tissue of the carrageenan-induced edema paw model, which include tumor necrosis factor-*α* (TNF-*α*), interleukin- (IL-) 1*β* and IL-6, NF-*κ*B, PGE_2_, NO, cyclooxygenase-2 (COX-2), and inducible nitric oxide synthase (iNOS), and evaluated the oxidative stress by quantifying the malondialdehyde (MDA) and myeloperoxidase (MPO) in the edema tissue and examining the activity of liver antioxidant enzymes involving superoxide dismutase (SOD), glutathione peroxidase (GPx), and glutathione reductase (GRd).

## 2. Materials and Methods

### 2.1. Plant Material

Flowers and buds of *C. indicum* were purchased from Qingping Chinese herbal medicine market of Guangzhou, Guangdong, China, and were identified by Professor Lai Xiao-Ping, School of Chinese Materia Medica, Guangzhou University of Chinese Medicine (GZUCM) in January 2012. The voucher specimen (number: VS-2012-1-09A01) was deposited at the Herbarium, GZUCM.

### 2.2. Chemicals

Carrageenan and Griess reagents were purchased from Sigma-Aldrich (St. Louis, USA). Evans blue, indomethacin (Indo), and Tween-80 were purchased from Sinopharm (Shanghai, CHN). Acetonitrile (HPLC grade) was purchased from Merck (Darmstadt, GER). Phosphate buffered saline (PBS) was purchased from Thermo Fisher Scientific (Beijing, CHN). Other reagents were of analytical grade.

### 2.3. Preparation of Plant Extract

The SCFE was conducted using a 532 supercritical fluid extract apparatus (Light Industry Institute of Guangzhou, Guangdong, CHN). Briefly, dry flowers and buds of *C. indicum* were exactly weighted and loaded into an extractor vessel. Based on the optimized extract process (data not shown), the total extract time was 4 h, the flow rate of CO_2_ was 20 L/h, the pressure was 25 MPa, and the temperature was 45°C with a modifier of 95.0% ethanol (10.0% of the sample weight). After removing the solvents, the oily CI_SCFE_ with a color of yellowish green (yield ratio was 5.142%, w/w) was collected. For pharmacological tests, the CI_SCFE_ was dissolved in 0.5% Tween-80 solution. To analyze the chemical composition of CI_SCFE_, the extract was partitioned between 75% methanol (100 mL × 3) and pure n-hexane (100 mL × 3) and condensed to a volume of 25 mL on a rotary evaporator. Compounds in pure n-hexane and 75% methanol layers were analyzed by GC-MS and HPLC-PAD, respectively.

### 2.4. Experimental Animals

Kunming (KM) mice (20–25 g) and Sprague Dawley (SD) rats (180–220 g) were obtained from the Laboratory Animal Services Centre of GZUCM. The animals were maintained on a 12 h light/a 12 h dark cycle under room temperature (22 ± 2°C) and humidity (50 ± 10%) and fed with standard forage and clean water *ad libitum*. All studies were conducted in accordance with the National Institutes of Health (NIH) Guide for the Care and Use of Laboratory Animals and used cervical dislocation to sacrifice animals. Except for the acute toxicity study, animals of either sex were divided into 5 or 6 groups (10 mice per group, 5 male and 5 female). Positive drug indomethacin (Indo) (10 mg/kg for mouse and 5 mg/kg for rat; p.o.) and CI_SCFE_ (40, 80, and 120 mg/kg for mouse; 20, 40, and 60 mg/kg for rat; p.o.) were given daily for 7 consecutive days. The control group was given an equal volume of 0.5% Tween-80 solution.

### 2.5. Chromatographic Analysis of CI_SCFE_

#### 2.5.1. GC-MS Analysis of CI_SCFE_

GC-MS analysis was carried out on an Agilent 6890-5975 GC-MS system consisting of an Agilent 6890 gas chromatography instrument, a 5975 mass spectrometer, and an Agilent ChemStation software (Agilent, Palo Alto, USA). Chromatographic separation was achieved on a 5% phenyl methyl siloxane HP-5MS capillary column (30 m × 0.25 mm inner diameter, 0.25 *μ*m film). The oven temperature was set initially at 60°C followed by a gradient of 10°C/min up to 100°C (held for 1 min) and then programmed to 110°C at 1°C/min (held for 1 min); furthermore, the temperature was up to 150°C at 3°C/min (held for 1 min) and finally to 260°C at 10°C/min (held for 5 min). Split injection (0.5 *μ*L) was conducted with a split ratio of 60 : 1, and helium was used as carrier gas of 1.0 mL/min flow rate. The spectrometer was set in electron-impact (EI) mode, the ionization energy was 70 eV, the scan range was 40–400 amu, and the scan rate was 0.34 s per scan. The inlet, ionization source temperatures were 230°C and 250°C, respectively. Identification of the compounds was based on a comparison of retention indices (relative to the retention times of n-alkanes on the HP-5MS column) and mass spectra with those of authentic samples, data from, the Wiley/NBS Registry of Mass Spectral Data (V.5.0), and the National Institute of Standards and Technology (NIST), and the MS Search (2011, V.2.0). The relative percentage of each compound in the n-hexane layer of CI_SCFE_ was quantified based on the peak area integrated by the analysis program.

#### 2.5.2. HPLC-PAD Analysis of CI_SCFE_

HPLC analysis was carried out on a Shimadzu LC-20A HPLC system consisting of a SPD-M20A PDA detector, a LC-20AT pump, a SIL-20AC automatic sampler, and a CTO-20A thermostatic column compartment (Shimadzu, Kyoto, Japan). The separation was performed on a Gemini C_18_ column (4.6 × 150 mm, 5 *μ*m, Phenomenex Inc., CA, USA) with a flow rate of 1.0 mL/min, column temperature at 30°C, and injection volume of 5 *μ*L. The mobile phase consisted of acetonitrile (solvent A), and 0.1% aqueous formic acid (solvent B) was used to elute the targets with the gradient mode (0–10 min: 5% A→15% A; 10–25 min: 15% A→30% A; 25–35 min: 30% A; 35–40 min: 30% A→50% A). Analysis based on the retention time and the ultraviolet (UV) absorption (190 to 800 nm) clearly authenticated the presence of chlorogenic acid, luteolin-7-glucoside, linarin, luteolin, and acacetin. The content of these compounds was quantitatively analyzed with peak areas under the standard curves at 334 nm.

### 2.6. Acute Toxicity Study

In compliance with the criterion previously described [22], KM mice were randomly divided into four groups (12 mice per group, 6 male and 6 female) to evaluate the acute toxicity of CI_SCFE_ after a single oral dose. The mice were administered orally with CI_SCFE_ (0.5, 1, 2, and 4 g/kg). All experimental animals were fed with standard diet and clean water *ad libitum* and kept under observation. The mortality or behavioral changes, including hyperactivity, tremors, ataxia, convulsions, salivation, diarrhea, lethargy, sleep, and coma, were regularly observed for 14 days. 

### 2.7. Xylene-Induced Mouse Ear Edema Model

This assay was performed according to the method as previously described [20, 23]. Briefly, 60 min after the last administration, each mouse received 20 *μ*L of xylene on the anterior and posterior surfaces of the right ear, and the left ear was considered as control. An hour later, animals were sacrificed and both ears were sampled with a punch (5 mm diameter) and weighted. The ear edema index was assessed as weight increase of the right ear over the left one.

### 2.8. Acetic Acid-Induced Mouse Vascular Permeability Model

 The test was carried out according to the method previously described with slight modifications [16, 20]. Briefly, 60 min after the last administration, each mouse was intravenously injected 0.5% Evans blue solution at 0.1 mL/10 g body weight followed by intraperitoneally injected 0.6% acetic acid at 0.1 mL/10 g body weight. Animals were sacrificed 30 min after acetic acid injection, and the peritoneal cavity was washed three times with a total of 4 mL of saline, which was combined and centrifuged for 10 min at 550 ×g. Supernatants were removed and measured at 590 nm by an Ultraviolet-visible Spectrophotometry (Shimadzu Co. Ltd., Kyoto, Japan). The amount of Evans blue extruded into the peritoneal cavity represented the capillary permeability.

### 2.9. Carrageenan-Induced Mouse Paw Edema Model

#### 2.9.1. Paw Edema Assay

The carrageenan-induced mouse paw edema test was conducted based on the method previously described with slight modification [20, 21, 24]. 60 min after the final administration, each mouse was given a subcutaneous injection of 25 *μ*L of 1% freshly prepared carrageenan suspension in saline into the plantar side of the right hind paw. The paw volume was measured before the injection as the basal volume (*V*_0_) and at intervals of 1–6 h after the chemical treatment as the pathological volume (*V*_1_) using the modified capillary amplification measurement. Percentage edema degree of paw edema was calculated using the following formula: % edema degree = (*V*_1_ − *V*_0_)/*V*_0_ × 100. Percentage inhibition of paw edema was calculated using the following formula: % inhibition = [Mean edema degree (control)/Mean edema degree (test)]/Mean edema degree (control) × 100.

To evaluate the effects of CI_SCFE_ on proinflammatory factors and oxidative stress, another set of mice was orally administered with 0.5% Tween-80, Indo, or CI_SCFE_. Four hours after the carrageenan induction, animals were sacrificed, and the right hind paws as well as the whole liver tissues were collected. The right hind paws were immediately placed in 9 volumes of cold PBS (v/w) and homogenized by using a TissueLyser II high-throughput tissue homogenization system (Qiagen Co. Ltd., Hilden, Germany). The homogenate was incubated on ice for 15 min and centrifuged at 10000 ×g for 15 min at 4°C. Then the supernatants were collected and stored at −80°C for later analysis of MPO, MDA, NO, TNF-*α*, IL-1*β*, IL-6, NF-*κ*B, COX-2, and iNOS. The liver tissue was immediately homogenized in cold PBS (1 : 1, v/w). The homogenate was centrifuged at 10000 ×g for 15 min at 4°C. The supernatants were collected and stored at −80°C for later analysis of antioxidant enzyme (SOD, GPx, and GRd) activities.

#### 2.9.2. MPO Assay

 The MPO activity was determined according to the method previously described [25, 26]. Each aliquot (5 mL) of supernatant was mixed with PBS (pH 7.4, 15 mL), NaH_2_PO_4_ (0.22 M, pH 5.4, 2 mL), H_2_O_2_ (0.026 [v/v] %, 2 mL), and tetramethylbenzidine (18 mM in 8% [v/v] aqueous dimethylformamide, 2 mL). After reacting for 10 min at 37°C following the adding of sodium acetate (1.46 M, pH 3.0, 3 mL), the enzyme activity of each reaction mixture was determined colorimetrically at 620 nm by using a Multiskan GO microplate spectrophotometer (Thermo Fisher Scientific, Waltham, Massachusetts, USA).

#### 2.9.3. NO Assay

 The content of nitrite as an indicator of NO production in the supernatant was measured according to the Griess reaction method described elsewhere [25]. Briefly, each supernatant was mixed with the same volume of Griess reagents. After incubation for 10 min at room temperature in dark place, the absorbance was measured at 540 nm using a microplate spectrophotometer.

#### 2.9.4. TNF-*α*, IL-1*β*, IL-6, and PGE_2_ Assay

The levels of TNF-*α*, IL-1*β*, IL-6, and PGE_2_ in the supernatants were measured using enzyme-linked immune sorbent assay (ELISA) kits (R&D Co. Ltd., Abingdon, UK) according to the manufacturer's instructions. Briefly, diluted standards or samples were added to 96-well plates precoated with affinity purified polyclonal antibodies specific for mouse TNF-*α*, IL-1*β*, IL-6, and PGE_2_, respectively. The wells were added with enzyme-linked polyclonal antibodies and incubated at 37°C for 60 min, followed by final washes for 5 times. The intensities detected at 450 nm were measured after addition of substrate solutions and were proportional to the productions of TNF-*α*, IL-1*β*, IL-6, and PGE_2_. 

#### 2.9.5. INOS and COX-2 Assay

The levels of iNOS and COX-2 in the supernatants were measured using ELISA kits (Cusabio Co. Ltd., WuHan, Hubei, China) according to the manufacturer's instructions. Briefly, process was the same as TNF-*α* assay described in Section 2.9.1.

#### 2.9.6. MDA and Antioxidant Enzymes Assay

The MDA concentration was determined using a commercially available MDA kit (Beyotime Institute of Biotechnology, Shanghai, China) according to the manufacturer's instructions. The activities of SOD, GPx, and GRd were measured using commercially available kits (Beyotime Institute of Biotechnology, Shanghai, China) according to the manufacturer's instructions, respectively.

### 2.10. Histological Analysis

Biopsies of right hind paws of mice were collected 4 h after the carrageen injection. Tissue slices were fixed in formalin-acetic acid fixative (10% formalin, 1% acetic acid) for 1 week at room temperature, dehydrated, embedded in paraffin, and sectioned into 4 *μ*m. Tissue sections were stained with hematoxylin and eosin (H&E stain) and examined with a BX60 microscope (Olympus Corporation, Kyoto, Japan). Images were captured with a Canon Power Shot G10 (Cannon Corporation, Shanghai, China). Every 3 to 5 tissue slices were randomly chosen from carrageen, Indo (10 mg/kg) and CI_SCFE_ (40, 80, and 120 mg/kg) groups. Paw swellings and enlarged cavities of every tissue slice were examined (10x and 100x).

### 2.11. Cotton Pellet-Induced Rat Granuloma Formation Model

The test was examined following previously described procedure with slight modification [20, 27]. Each rat was anaesthetized with 10% chloral hydrate (1 mL/kg), and an incision was made in the lumbar region, then a sterilized cotton pellet (100 ± 1 mg) was inserted into the subcutaneous tunnel on the day before drug administration. After the final administration, the rats were anaesthetized by 10% chloral hydrate, and pellets together with the granuloma tissues were removed surgically and separated from extraneous tissues. Pellets were dried at 60°C until a constant weight was obtained, and the net dry weight of granuloma was compared to evaluate the granuloma tissue formation.

### 2.12. Statistical Analysis

All data represented the mean ± SEM. Statistical analyses were performed with SPSS 18.0 software and were carried out using one-way ANOVA followed by Scheffe's multiple range test.

## 3. Results

### 3.1. Chromatographic Analysis of CI_SCFE_

#### 3.1.1. GC-MS Analysis

**Figure 1:**
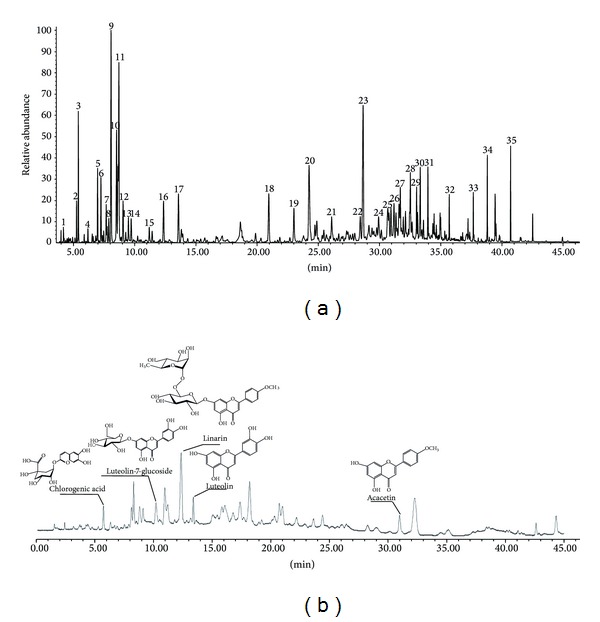
GC-MS and HPLC chromatographs of CI_SCFE_. (a) GC-MS chromatographs of the n-hexane layers of CI_SCFE_. (b) HPLC chromatographs of the 75% methanol layers of CI_SCFE_.

A total of 35 main ingredients constituting 89.709% of the n-hexane layer of CI_SCFE_ were identified as shown in Table 1. The chemical profile is presented in Figure 1(a). The principal components were d-camphor (8.582%), caryophyllene oxide (8.460%), endo-borneol (7.845%), *α*-curcumene (5.932%), cis-verbenol (4.720%), *β*-caryophyllene (3.336%), eucalyptol (3.091%), thymol (3.071%), and bornyl acetate (2.948%).

#### 3.1.2. HPLC-PAD Analysis

The HPLC analysis profile (334 nm) of 75% methanol layer of CI_SCFE_ was showed in Figure 1(b), and the relative amounts of five verified compounds are chlorogenic acid (21.1 mg/g), luteolin-7-glucoside (22.8 mg/g), linarin (48.3 mg/g), luteolin (11.4 mg/g), and acacetin (8.8 mg/g).

### 3.2. Acute Toxicity Study

After 14 days of a single oral dose, CI_SCFE_ did not cause any behavioral changes, and no mortality was observed. Therefore, the maximal tolerance dose (MTD) of CI_SCFE_ was supposed to be larger than 4 g/kg in mice.

### 3.3. Effect of CI_SCFE_ on Xylene-Induced Mouse Ear Edema

**Figure 2:**
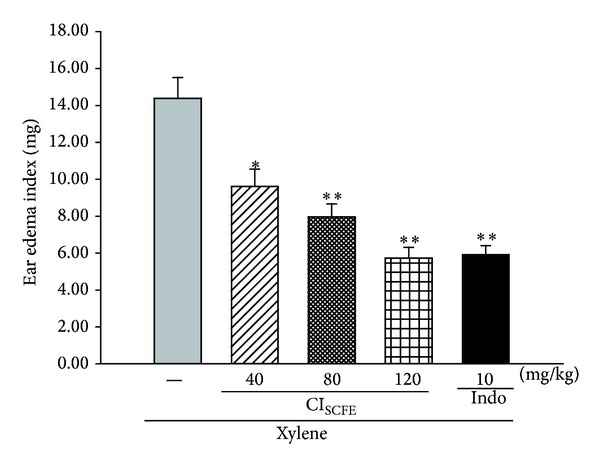
Inhibitory effect of CI_SCFE_ on the xylene-induced ear edema. The ear edema index was represented as the mean ± SEM (*n* = 10). *P < 0.05 and **P < 0.01 compared to the xylene-treated control group.

Application of xylene in mouse ears brought about a significant increase of ear weight in the xylene-treated control group (Figure 2). In contrast, compared with the xylene-treated control group, animals in CI_SCFE_ (40, 80, and 120 mg/kg) groups showed significant inhibitions of ear edema at 33.21%, 44.68%, and 60.18%, respectively (for all, *P* < 0.05). The results demonstrated that CI_SCFE_ dose dependently decreased the xylene-induced mouse ear edema. In addition, the inhibition of CI_SCFE_ at 120 mg/kg on ear edema was approximate to Indo (for both, *P* < 0.01 versus xylene-treated control). 

### 3.4. Effect of CI_SCFE_ on Acetic-Acid-Induced Vascular Permeability

**Figure 3:**
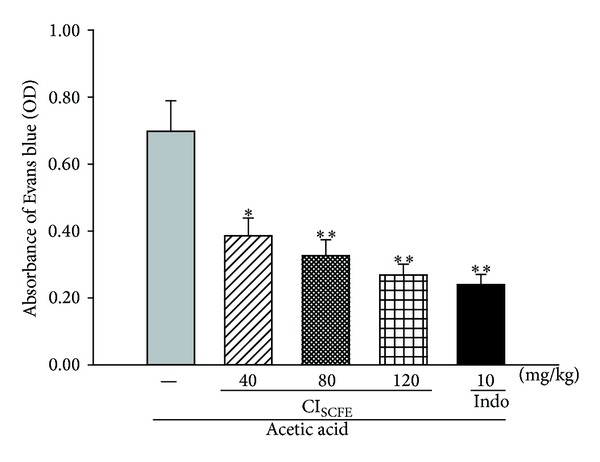
Inhibitory effect of CI_SCFE_ on the capillary permeability under acetic acid challenge. The absorbance of Evans blue in the leakage under 590 nm was represented by the mean ± SEM (*n* = 10). *P < 0.05 and **P < 0.01 compared to the acetic acid-treated control group.

 The capillary permeability was represented by the amount of Evans blue extruded into peritoneal cavity, which was measured by the OD of the supernatant. In the current study, the OD of the acetic acid-treated control group markedly increased under the challenge of acetic acid (Figure 3). When compared with the acetic acid-treated control group, CI_SCFE_ (40, 80 and 120 mg/kg) significantly produced a dose-dependent inhibitory effect on the OD at 44.67%, 53.21%, and 62.98%, respectively (for all, *P* < 0.05). These data showed that CI_SCFE_ markedly attenuated acetic acid-induced capillary permeability.

### 3.5. Effect of CI_SCFE_ on Carrageenan-Induced Mouse Paw Edema

**Figure 4:**
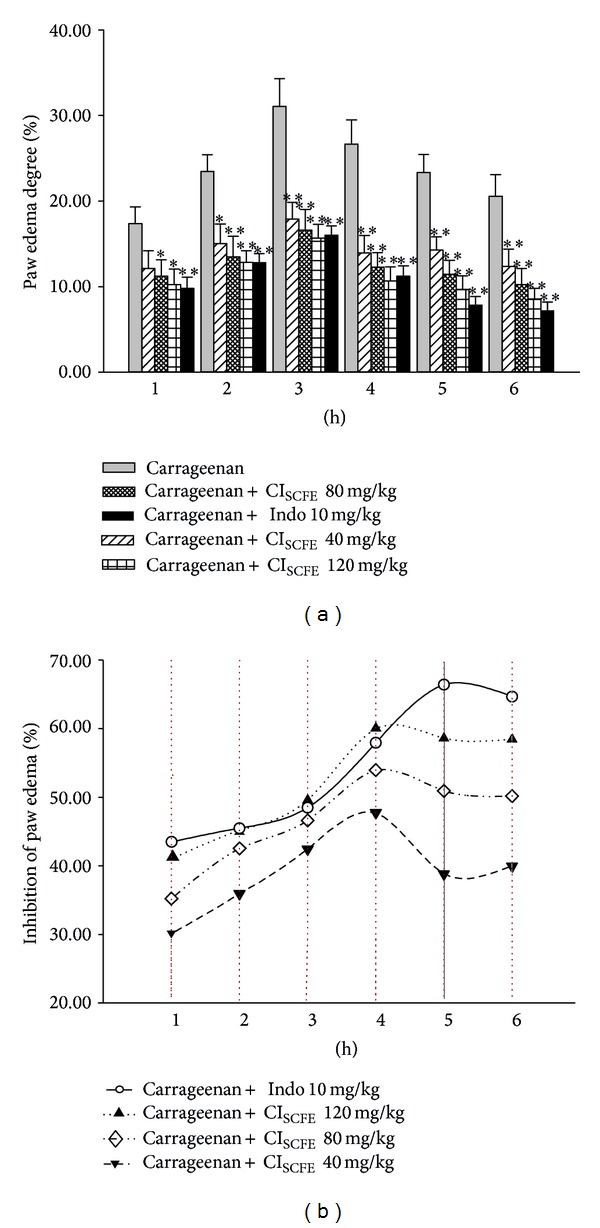
Inhibitory effect of CI_SCFE_ on the carrageenan-induced mouse paw edema. (a) Paw edema degree was represented as the mean ± SEM (*n* = 10). (b) Suppression of paw edema (%) was represented as the ratio of the mean paw size increase of drug treatment group (%) on that of the carrageenan-treated control group (%). *P < 0.05 and **P < 0.01 compared to the carrageenan-treated control group.

 In this test, the edema was measured by the volume of paw and indicated the inflammatory activity. As Figure 4 shows, after the carrageenan treatment, the paw volume of the carrageenan-treated control group obviously increased in a time-dependent manner. When compared with the carrageenan-treated control group, the CI_SCFE_ (40, 80, and 120 mg/kg) significantly suppressed paw edema increase dosedependently at the 2nd to 6th hour (for all, *P* < 0.05) after carrageenan induction (Figure 4(a)). The peak paw edema inhibition of CI_SCFE_ was presented at the 4th hour (for all, *P* < 0.05 versus carrageenan-treated control) in a dose-dependent manner and then gradually decayed as time went by (Figure 4(b)). CI_SCFE_ at the dose of 120 mg/kg showed almost equal amount of inhibition as 10 mg/kg Indo (for both, *P* < 0.01 versus carrageenan-treated control) (Figure 4(b)).

#### 3.5.1. Effect of CI_SCFE_ on MPO Level

**Figure 5:**
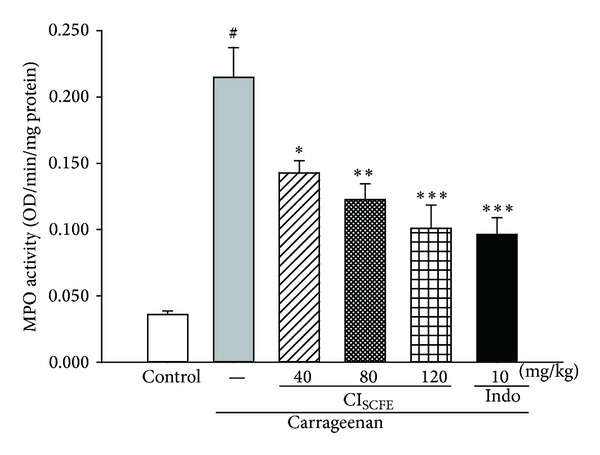
Effect of CI_SCFE_ on carrageenan-induced activity of MPO in mouse paw edema. Data represented the mean ± SEM (*n* = 10). *P < 0.05 and **P < 0.01 compared to the carrageenan-treated control group.

MPO is a common index of inflammation [26]. As Figure 5 shows, when compared to the control group, the MPO activity of the carrageenan-treated control group was significantly increased (*P* < 0.01). However, treating the animals with CI_SCFE_ (40, 80, and 120 mg/kg) markedly suppressed the MPO activity by 33.55%, 43.01%, and 53.05% (for all *P* < 0.05 versus carrageenan-treated control), respectively. The suppression on the MPO activity indicated that the inflammatory response was attenuated.

#### 3.5.2. Effect of CI_SCFE_ on TNF-*α*, IL-1*β*, IL-6, and NF-*κ*B Levels

**Figure 6:**
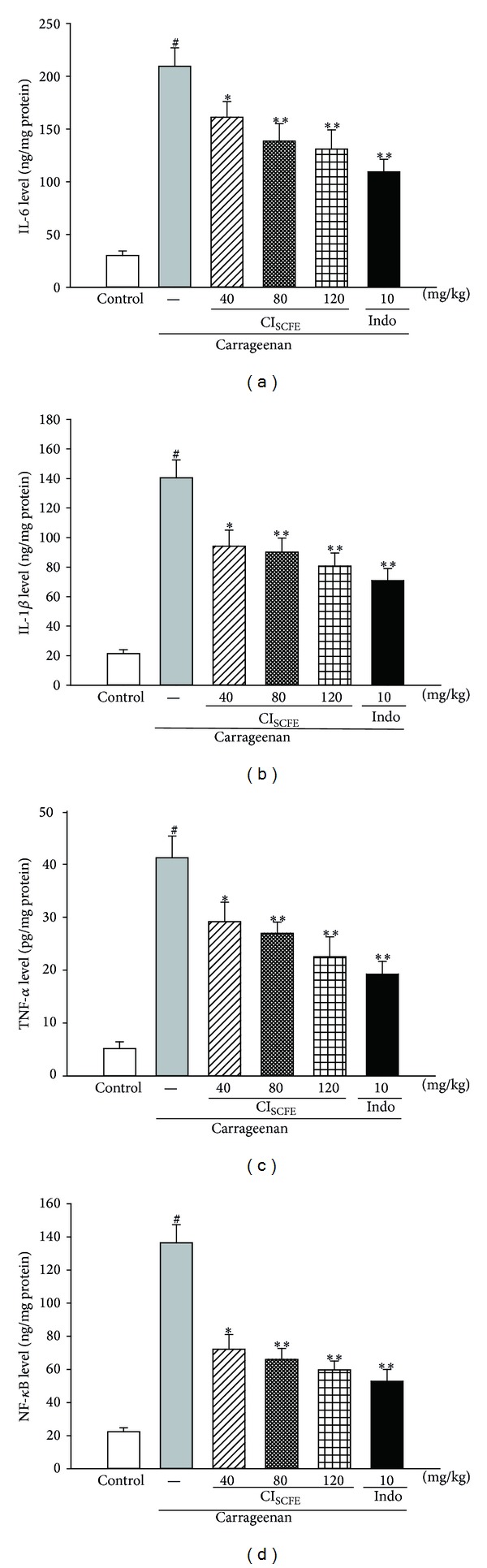
Effect of CI_SCFE_ on carrageenan-induced level of IL-6 (a), IL-1*β* (b), TNF-*α* (c), and NF-*κ*B (d) in mouse paw edema. Data represented the mean ± SEM (*n* = 10). ^#^ *P* < 0.01 compared to the control group; *P < 0.05 and **P < 0.01 compared to the carrageenan-treated control group.

When compared with the control group, the protein levels of TNF-*α*, IL-1*β*, IL-6, and NF-*κ*B in carrageenan-induced edema paws of the carrageenan-treated control group were remarkably raised (Figure 6). In terms of TNF-**α**, CI_SCFE_ (40 and 80 mg/kg) had an inhibitory effect on it as compared to the carrageenan-treated control group (both *P* < 0.05) despite that the low dose of 40 mg/kg did not reduce the TNF-**α**level statistically. In addition, CI_SCFE_ (40, 80, and 120 mg/kg) dose dependently downregulated the protein level of IL-1*β*, IL-6, and NF-*κ*B as compared to the carrageenan-treated control group (for all, *P* < 0.05).

#### 3.5.3. Effect of CI_SCFE_ on NO and iNOS Levels

**Figure 7:**
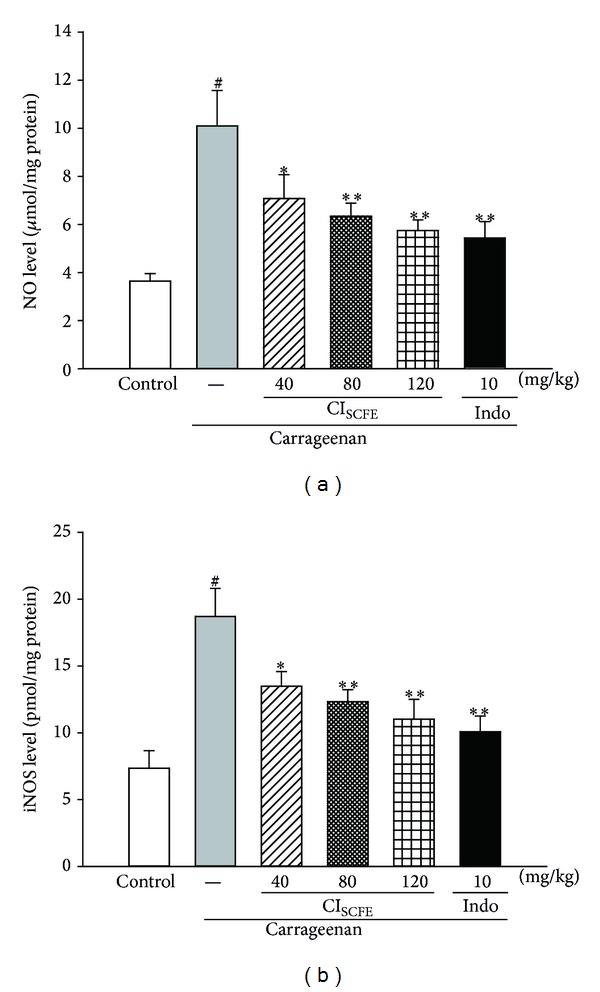
Effect of CI_SCFE_ on carrageenan-induced level of NO (a) and iNOS (b) in mouse paw edema. Data represented the mean ± SEM (*n* = 10). ^#^ *P* < 0.01 compared to the control group; *P < 0.05 and **P < 0.01 compared to the carrageenan-treated control group.

The effect of CI_SCFE_ on the NO production and the iNOS level was shown in Figure 7. Carrageenan treatment increased the production of NO and the level of iNOS of the carrageenan-treated control group (for both *P* < 0.05 versus control group). However, CI_SCFE_ (40, 80, and 120 mg/kg) significantly decreased the levels of NO and iNOS dose dependently (for all, *P* < 0.05 versus carrageenan-treated control). 

#### 3.5.4. Effect of CI_SCFE_ on PGE_2_ and COX-2 Levels

**Figure 8:**
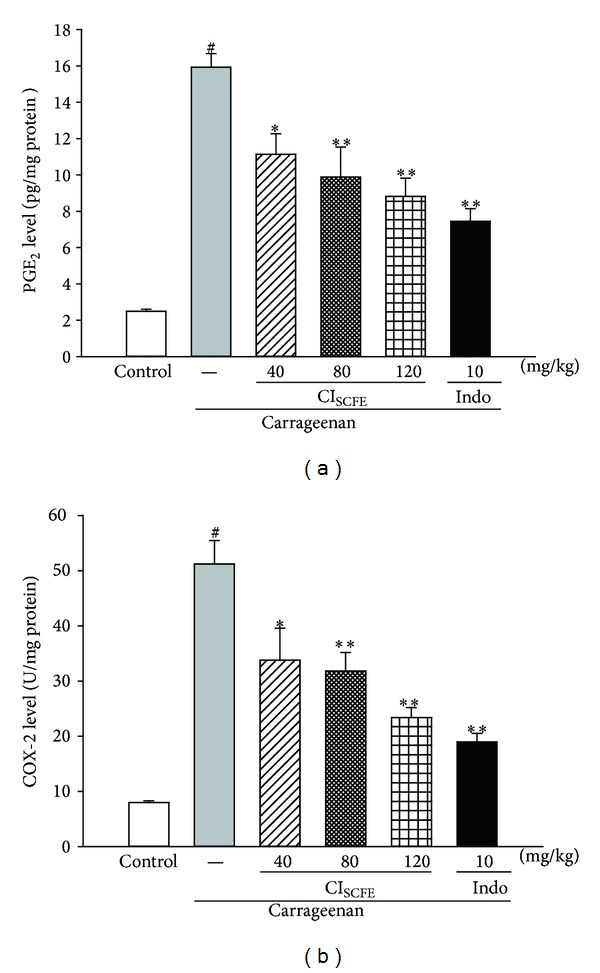
Effect of CI_SCFE_ on carrageenan induced the level of PGE_2_ (a) and COX-2 (b) in mouse paw edema. Data represented the mean ± SEM (*n* = 10). ^#^ *P* < 0.01 compared to the control group; *P < 0.05 and **P < 0.01 compared to the carrageenan-treated control group.

 The effect of CI_SCFE_ on the PGE_2_ production and the COX-2 level was shown in Figure 8. Carrageenan treatment increased the levels of PGE_2_ and COX-2 of the carrageenan-treated control group, compared to those of the control group (for both *P* < 0.05). However, the levels of NO and iNOS in the CI_SCFE_ (40, 80, and 120 mg/kg) groups were significantly suppressed in a dose-dependent manner, as compared to the carrageenan-treated control group (for all, *P* < 0.05).

#### 3.5.5. Effect of CI_SCFE_ on MDA Level

In the carrageenan-treated control group, MDA level in the carrageenan-induced edema paw remarkably increased, compared to the control group (Table 2). However, the MDA level decreased significantly with treatment of CI_SCFE_ (40, 80, and 120 mg/kg) as well as 10 mg/kg Indo (for all, *P* < 0.05 versus carrageenan-treated control).

#### 3.5.6. Effect of CI_SCFE_ on Antioxidant Enzymes' Levels

 The activities of SOD, GPx, and GRd at the 4th h following the intrapaw injection of carrageenan in carrageenan-treated control group decreased significantly compared to the control group (*P* < 0.01), as presented in Table 2. However, compared to the carrageenan-treated control group, pretreatments with CI_SCFE_ (40, 80, and 120 mg/kg) boosted the SOD, GPx, and GRd activities significantly (for all *P* < 0.001).

### 3.6. Histopathological Analysis

**Figure 9:**
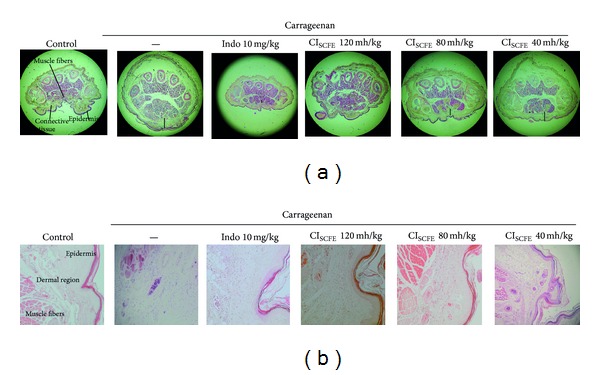
Histopathological examinations on carrageenan-induced paw tissue swelling, edema, haemorrhage, and leukocyte infiltration. (a) The level of inflammatory response was represented as the distance of swelling connective tissue (10x). (b) The level of inflammatory response was represented as the level of haemorrhage (red blood cells) and leukocyte infiltration (neutrophil infiltration) of the dermis region (100x).

 As it is shown in Figure 9(a), no inflammation, tissue destruction, or swelling phenomenon was observed in the paws of the control group. On the other hand, carrageenan-treated control group displayed enlarged cavities. As for the positive control group and experimental groups, an edematous condition was obviously abated by treatment with Indo (10 mg/kg) and CI_SCFE_ (40, 80, and 120 mg/kg).  Figure 9(b) showed marked cellular infiltration in the connective tissue of the carrageenan-treated control group, and the infiltrates accumulated between collagen fibres and in intercellular space. However, when compared to the carrageenan-treated control group, CI_SCFE_ (40, 80, and 120 mg/kg) showed a reduction in carrageenan-induced inflammatory response. Inflamed cells and cellular infiltration were decreased. Collagen fibres were regular in shape and showed reductions in intercellular spaces.

### 3.7. Effect of CI_SCFE_ on Cotton Pellet-Induced Rat Granuloma Formation

**Figure 10:**
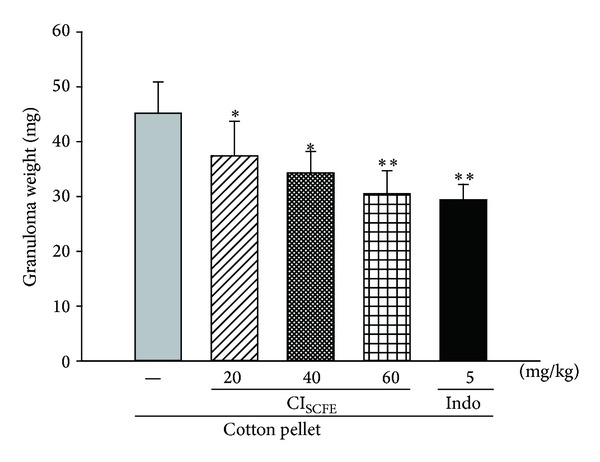
Inhibitory effect of CI_SCFE_ on the cotton pellet-induced granuloma formation. Data represented the mean ± SEM (*n* = 10). *P < 0.05 and **P < 0.01 compared to the cotton pellet-treated control group.

 In this model, granuloma weight is an index of granuloma tissue formation, which is used to evaluate the chronic inflammation. As shown in Figure 10, cotton pellet induced granuloma formation of the cotton pellet-treated control group. CI_SCFE_ groups (especially the 80 and 120 mg/kg groups) exhibited significant inhibitions on the formation of granuloma. In details the mean granuloma weight of CI_SCFE_ group at the dose of 120 mg/kg was reduced significantly (*P* < 0.01 versus cotton pellet-treated control). This effect was almost equally potent as Indo (5 mg/kg) (for both *P* < 0.01 versus cotton pellet-treated control). 

## 4. Discussion

In this study, to investigate whether CI_SCFE_ has an anti-inflammatory activity, we evaluated the effect of CI_SCFE_ in four animal models, including xylene-induced mouse ear edema, acetic acid-induced mouse vascular permeability, carrageenan-induced mouse hind paw edema, and cotton pellet-induced rat granuloma formation. To clarify the chemical composition of CI_SCFE_, we firstly analyzed the CI_SCFE_ by combining GC-MS with HPLC-PAD. 

In the process of inflammation, vasodilatation brings about plasma extravasations and inflammatory mediator releases, which trigger the acute inflammation response [16–18, 20]. In xylene-induced mouse ear edema assay, treatment with CI_SCFE_ (40–120 mg/kg) significantly decreased the ear edema in a dose-dependent manner. The inhibition of ear edema indicated that CI_SCFE_ attenuated vasodilatations and plasma extravasations of neurogenic inflammation, which are crucial in controlling the early stage of acute inflammation.

In acetic acid-induced vascular permeability test, acetic acid challenge brings about increases in the level of mediators such as prostaglandin, serotonin, and histamine in peritoneal fluids, which in turn lead to a dilation of the capillary vessels and an increase in vascular permeability [16–18, 20]. Experimental data showed that CI_SCFE_ (40, 80, 120 mg/kg) dose dependently attenuated the capillary permeability accentuation induced by acetic acid in mice. Therefore, this result consolidates that the anti-inflammatory effect of CI_SCFE_ in the acute phase of inflammation associates with prevention of vasodilation and may be mediated by inhibiting the releases of inflammatory mediators.

The carrageen-induced mouse paw edema is a reliable and repeatable model for evaluating the anti-inflammatory effect of natural products [20, 21, 24]. Consistent with the above results, histopathological examination demonstrated that CI_SCFE_ (even at a lowest dose of 40 mg/kg) significantly improved edematous conditions and decreased intracellular spaces induced by carrageenan, showing that CI_SCFE_ downregulated the carrageenan-induced inflammatory response in mice. 

The development of carrageen-induced paw edema is commonly characterized as a biphasic event [20, 21]. The first phase of edema (0–2 hours) is mediated by histamine and serotonin followed by kinin and finally through bradykinin, PGs, and lysosome [20, 21]. The late phase (2–6 hour) is to be correlated with the enhanced production of NO, PGs, TNF-*α*, IL-1*β*, and IL-6, which could in turn worsen the level of inflammatory response [20, 21]. NF-*κ*B, TNF-*α*, IL-1*β*, and IL-6 are involved in neutrophil migration in carrageenan-induced inflammation [28–31]. These mediators are able to recruit leukocytes, such as neutrophils, as reported in several recent experimental models [28–31]. In the current study, CI_SCFE_ significantly inhibited carrageenan-induced inflammatory response and acted more effectively in the second phase of inflammation than in the first phase. MPO is a common *in vivo* index of granulocyte infiltration and inflammation and is a marker of oxidative stress [25, 26]. Therefore, the MPO activity of carrageen-induced paw edema in mice was measured. Results demonstrated that CI_SCFE_ markedly suppressed MPO activity in a dose-dependent manner, indicating that inflammation in carrageenan-induced was alleviate. Moreover, the levels of NF-*κ*B, TNF-*α*, IL-1*β*, and IL-6 were also decreased by treatment with CI_SCFE_ and Indo. Thus, the anti-inflammatory mechanism of CI_SCFE_ may be associated with inhibition on inflammatory mediators such as NF-*κ*B, TNF-*α*, IL-1*β*, and IL-6.

COX-2 selective inhibitor is a form of NSAID that directly targets COX-2, an enzyme responsible for inflammation [32]. Similarly, iNOS generates NO and has been regarded as having a central role in inflammatory responses [19, 33]. Inhibition of NO and PGE_2_ productions via suppressing iNOS and COX-2 expressions is beneficial in treating inflammatory diseases [34]. It is commonly known that the expression of COX-2 and iNOS is maximal at the late phase of carrageenan-induced paw edema [20, 21, 35]. Our study demonstrated that the CI_SCFE_ downregulated iNOS and COX-2 protein expressions, suggesting that the anti-inflammatory effect of CI_SCFE_ may be related to the inhibition on PGE_2_ and NO syntheses. This putative mechanism is similar to that of Indo, the drug used as the positive control, which mediated anti-inflammation via inhibiting PGE_2_ and NO pathways in carrageenan-induced inflammation [36]. 

Previous researchers have demonstrated that the carrageenan-induced inflammatory response is linked to neutrophil infiltration and the production of reactive oxygen species (ROS) [19, 37]. ROS such as hydrogen peroxide (H_2_O_2_), superoxide anion radicals (O_2_^•−^), and hydroxyl radicals (OH^•^) play major roles in terms of producing cellular damage in inflammatory processes [38]. MDA, produced via free radical attack on the plasma membrane, forms covalent protein adducts. Thus, accumulation of MDA resulted from the inflammatory effect which manifests the degree of inflammation. ROS have been proposed to mediate cell damage via the inactivation of antioxidant defense systems. A variety of antioxidant enzymes, such as SOD, GPx, and GRd, scavenge and minimize the formation of ROS [39]. SOD protects cells against the damages of ROS. GPx, in the presence of Glutathione (GSH), accelerates the reduction of H_2_O_2_ or other organic hydroperoxides and serves as a second line of defense against hydroperoxides [40]. GRd plays a crucial role in cellular defense against oxidative stress by preventing accumulation of oxidized glutathione (GSSG), thus maintaining the redox state. In this test, there was a significant decrease in the level of MDA after CI_SCFE_ application. On the other hand, CI_SCFE_ significantly enhanced the activities of SOD, GPx, and GRd in the liver. These results showed that CI_SCFE_ exhibited a positive regulation of antioxidative activities against inflammatory oxidation. The suppression of MDA production is probably due to the activation of antioxidant enzymes including SOD, GPx, and GRd. 

In addition, we evaluated the anti-inflammatory activity of CI_SCFE_ on chronic inflammation. The induction of granuloma formation by inserting cotton pellet subcutaneously into a rat is widely used to study the transudative and proliferative phases of chronic inflammation [20, 27]. The results of cotton-pellet granuloma formation model demonstrated that CI_SCFE_ inhibited the formation of granuloma in chronic inflammation.

In the current study, we firstly analyzed the chemical composition by combining GC-MS with HPLC-PAD. Based on the GC-MS results, the 35 identified main constituents can be classified into monoterpenes (e.g., eucalyptol and bornyl acetate), sesquiterpenes (e.g., *β*-caryophyllen and caryophyllene oxide), phenolic compounds (e.g., thymol and *α*-bisabolol oxide), and alkenes (e.g., *α*-curcumene and cis-verbenol). Most of these compounds have been identified in plant essential oils and exhibit significant antioxidative activities against inflammation via scavenging ROS and inhibiting leukocyte migration [41, 42]. Previous studies revealed that eucalyptol, thymol, d-camphor, and endo-borneol can promptly and dose dependently downregulate proinflammatory mediators such as TNF-*α*, IL-1*β*, and IL-6 and increase the anti-inflammatory cytokine IL-10 [42, 43]. In addition, by HPLC-PAD analysis, we identified and quantified 5 components: chlorogenic acid, luteolin-7-glucoside, linarin, luteolin, and acacetin. Chlorogenic acid can inhibit ROS production and block NF-*κ*B signaling, which result in significant antioxidative and anti-inflammatory properties [5, 6, 9–13]. Flavonoids, such as luteolin-7-glucoside, linarin, luteolin, and acacetin, also possess significant anti-inflammatory properties, partly related to the inhibition on iNOS and COX-2 expressions in activated macrophages [5, 6, 9–13]. Thus, the chemical analysis of CI_SCFE_ suggested that these major components are possibly responsible for the regulation of inflammatory factors against inflammation response.

## 5. Conclusion 

This paper firstly analyzed the chemical composition of CI_SCFE_ by combining GC-MS with HPLC-PAD. Thirty-five compounds were identified by GC-MS, and five compounds with anti-inflammatory activity, chlorogenic acid, luteolin-7-glucoside, linarin, luteolin, and acacetin were reconfirmed and quantified by HPLC-PAD. This study systematically investigated the anti-inflammatory property of CI_SCFE_. The result provides the first evidence for the anti-inflammatory application of CI_SCFE_ and suggests which mechanism may relate to antioxidation and regulation of inflammatory factors. 

## Figures and Tables

**Table 1:** Chemical compositions of the n-hexane layers of CI_SCFE_ by GC-MS analysis.

Number	Components	R.t.^a^	K.I.^b^	Percentage (%)^c^
1	Camphene	4.18	818.6	0.475
2	*β*-Cymene	5.25	1027.5	0.998
3	Eucalyptol	5.40	1056.6	3.091
4	Linalool oxide	6.19	1128.3	0.521
5	*α*-Thujone	6.97	1169.2	2.186
6	*β*-Thujone	7.24	1183.0	2.169
7	Isothujol	7.68	1204.5	1.094
8	L-Pinocarveol	7.89	1214.6	0.765
9	d-Camphor	8.07	1223.4	8.582
10	Cis-verbenol	8.53	1244.6	4.720
11	endo-Borneol	8.72	1253.5	7.845
12	L-4-Terpineol	9.06	1269.6	1.634
13	*α*-Terpineol	9.49	1289.5	1.022
14	Myrtenol	9.72	1300.4	1.054
15	Cuminaldehyde	11.43	1353.1	0.486
16	Bornyl acetate	13.59	1411.0	2.948
17	Thymol	13.81	1415.5	3.071
18	*β*-Caryophyllen	20.97	1548.8	3.336
19	cis-*β*-Farnesene	23.02	1587.9	2.270
20	*α*-Curcumene	24.26	1615.7	5.932
21	*δ*-Cadinene	26.10	1663.1	1.815
22	Spathulenol	28.44	1728.0	1.362
23	Caryophyllene oxide	28.67	1735.2	8.460
24	*γ*-Eudesmol	29.93	1774.3	1.568
25	T-Muurolol	31.19	1815.1	1.487
26	*α*-Gurjunene	31.60	1832.5	2.161
27	Aromadendrene	31.71	1834.5	2.280
28	*α*-Bisabolol	32.53	1848.9	2.289
29	Cubenol	33.04	1880.1	1.742
30	Longifolenaldehyde	33.34	1890.6	2.572
31	*α*-Bisabolol oxide	33.96	1913.3	2.600
32	Hexahydrofarnesyl acetone	35.70	1981.3	1.212
33	Ethyl hexadecanoate	37.67	2069.6	1.362
34	*α*-Linolenic acid	38.82	2129.8	2.130
35	Ethyl octadec-9,12-dienoate	39.45	2145.0	2.470

^a^Retention time (min).

^b^Kovats index relative to n-alkanes (C_6_–C_30_) on HP-5MS column.

^c^Relative percentage calculated by integrated peak area in Agilent MSD Chemstation data analysis program.

**Table 2:** Effect of CI_SCFE_ on carrageenan-induced level of MDA in mouse paw edema and the activities of SOD, GPx, and GRd in mouse liver.

Groups	MDA (nmol/mg protein)	SOD (U/mg protein)	GPx (U/mg protein)	GRd (U/mg protein)
Control	0.237 ± 0.093	10.247 ± 0.364	0.275 ± 0.028	2.365 ± 0.302
Carrageenan	1.391 ± 0.096^#^	3.811 ± 0.201^#^	0.087 ± 0.009^#^	0.422 ± 0.090^#^
Carrageenan + CI_SCFE_ (40 mg/kg)	0.593 ± 0.042**	5.736 ± 0.285**	0.158 ± 0.013*	1.268 ± 0.125**
Carrageenan + CI_SCFE_ (80 mg/kg)	0.467 ± 0.043**	6.258 ± 0.223**	0.182 ± 0.022*	1.594 ± 0.178**
Carrageenan + CI_SCFE_ (120 mg/kg)	0.432 ± 0.032**	7.305 ± 0.267**	0.217 ± 0.027**	1.755 ± 0.167**
Carrageenan + Indo (10 mg/kg)	0.482 ± 0.035**	6.482 ± 0.159**	0.185 ± 0.019**	1.617 ± 0.144**

Data represented the mean ± SEM (*n* = 10).  ^#^ *P* < 0.01 compared to the control; *P < 0.05 and **P < 0.01 compared to the carrageenan-treated control group.
